# An assessment of the performance of the Chemispek J200 in a hospital laboratory

**DOI:** 10.1155/S1463924682000315

**Published:** 1982

**Authors:** Gordon Dale, Andrew McGill, David R. Walton

**Affiliations:** Department of Clinical Biochemistry Newcastle General Hospital Westgate Road Newcastle upon Tyne NE4 6BE UK


Journal of Automatic Chemistry, Volume 4, Number 3 (July–September 1982), pages 122–124

### An assessment of the performance of the Chemispek J200 in a hospital laboratory

**Gordon Dale, Andrew McGill and David R. Walton**

*Department of Clinical Biochemistry, Newcastle General Hospital, Westgate Road, Newcastle upon Tyne NE4 6BE, UK*

### Introduction

The Chemispek J200 (Hilger Analytical, Westwood, Margate, Kent CT9 4JL, UK) is a recently introduced continuous-flow multichannel autoanalyser allowing up to 16 simultaneous analyses. In its six-parameter configuration 140 $\mu$l of sample is analysed at 120 specimens/h. This report summarizes its performance using 'PROM 6' in the microprocessor.

### Equipment

The Chemispek comprises several interconnecting modules: sampler, gear-box and pumps, reagent valves, chemistry platforms, photometers, flame photometer, recorder, microprocessor, printer, cassette-recorder and power pack.

(1) The sampler is electronically variable for both wash and sample time. The 60-position sample plate is removable.
(2) Each pump and gear-box serves a pair of analytical platforms. Each pump has four speeds. There is one operating speed (5 rev./min.), one fast and two slow speeds.
(3) The reagent valves have three positions, reagent, wash and a clamped position to prevent reagents running back. Each analytical platform is controlled by its own set of reagent valves.
(4) There is an analytical platform for each chemistry, except sodium and potassium. The platform contains a special block to facilitate the introduction of air bubbles into the liquid phase in a uniform manner. Mixing coils, dialysers, heating bath and connecting tubing are all located on the platform. The waste from the flow cell drains away under the platform where it is piped into a central drain.
(5) The twin-channel photometer incorporates a quartz iodine light-source and an interference filter for each channel. Each channel has its own circuitry to allow for normal or inverse chemistry, blank correction mode and curve regeneration. The photometer uses a special 5mm flow cell which has angled reflective glass surfaces at each end. This enables the photometer to use an energy gating system to measure the optical density of the liquid phase without the need to debubble.
(6) A flame photometer with integral diluter (Corning 455) is connected to the microprocessor via a digital analogue converter. The sample is obtained using a split-stream connector connected to the sample line.
(7) A Bryans Southern six-pen recorder is used to monitor the six channels (1/6 full-scale deflection or full-scale deflection). Each channel can be controlled independently for scale deflection.
(8) A 16-channel microprocessor is used to monitor the signals from all photometers. Each chemistry is assigned its own microprocessor channel which standardizes, calculates and corrects for carry-over and drift. The results for each channel are then printed out along with identification of sample number, run number, date, table heading and a flagging system for abnormal peaks and results which lie outside an assigned reference range.
(9) A command terminal/printer (Digital decwriter II) is used to print the results as well as to communicate with the microprocessor.
(10) A cassette tape-recorder is used to record the data stored in the microprocessor after standardization. These data may be quickly loaded back into the microprocessor when necessary.
(11) A power unit with a fuse board is supplied.

### Methods

*Chemistry*

The methods used are common on Technicon SMA autoanalysers and are summarized in table 1. The reagents used were either bought in ready-made, or made up with chemicals obtained from BDH (BDH Chemicals Ltd, Broom Road, Poole, Dorset, UK).

*Table 1. Chemistry methods used on Chemispek J200.*

| | Method | Reference |
|---|---|---|
| Sodium | Corning 455 photometry | |
| Potassium | Corning 455 photometry | |
| Chloride | Thiocyanate—nitrate | Skeggs and Hochstrasser [1] |
| Bicarbonate | Cresol red | Skeggs and Hochstrasser [1] |
| Urea | Diacetyl monoxime | Marsh *et al.* [2] |
| Creatinine | Alkaline picrate | Chasson *et al.* [3] |

*Standardization*

The standard plate allows for up to seven standards to be used. Five aqueous standards were prepared with concentrations as shown in table 2. The photometer outputs of these standards in millivolts were obtained on the printer and were compared graphically and by regression analysis. In addition to aqueous standards, four commercial sera were obtained with the concentrations as shown in table 3 (General Diagnostics: three sera, Wellcome Reagents Ltd: one serum) to assess their suitability as secondary standards. The photometer outputs of these sera were obtained on the printer and compared graphically and by regression analysis.

*Carry-over correction*

At the start of the evaluation period the carry-over correction factors were calculated by the microprocessor from duplicate standards. Later, the microprocessor was updated and carry-over correction factors were calculated from a carry-over plate with the following composition:

 0142-0453/82/0403 0122 $05·00 © 1982 Taylor & Francis Ltd

Drift control: cup 1.
High-concentration standard: cups 2,3,4,8,9,10.
Low-concentration standard: cups 5,6,7,11,12,13.

The carry-over correction of serum samples were then assessed using the method described by Broughton *et al.* [4].

*Drift correction*

A drift control was placed at positions 2,11,12,21,22,31,32,41,42, 51,52,59,60 on the 60-position plate. A comparison of test results using 'no drift correction' against 'drift correction' showed that there was a marked improvement of test results when 'drift correction' was used.

*Curve regeneration*

Curve regeneration settings were investigated on the four colorimetric channels (chloride, bicarbonate, urea, creatinine). The changes in carry-over effect for each curve regeneration setting was assessed for serum samples using the method of Broughton *et al.* [4].

*Within-run imprecision*

Within-run imprecision was determined using the protocol described by McLelland *et al.* [5]. Three sera (low, medium and high concentration) were used and two sample plates were prepared with the following composition:

Drift standard:
position 1,2,11,12,21,22,31,32,39,40.
Low concentration sera:
position 4,9,14,15,19,24,27,28,34,37.
Medium concentration sera:
position 6,7,10,16,18,20,23,26,29,33.
High concentration sera:
position 3,5,8,13,17,25,30,35,36,38.

The results were corrected for both drift and carry-over.

*Between-run imprecision*

Between-run imprecision was determined by analysing two commercial sera at the beginning of each run. The two sera were assayed in a total of 107 runs over a four-week period.

*Inaccuracy*

Lyophilized material supplied for the Wellcome quality assurance scheme and the National quality assurance scheme were analysed on the same day that the material was reconstituted. The results obtained were then compared with the consensus value obtained from the quality-control files.

### Results

*Standardization*

Using either aqueous standards or secondary standards four channels were linear through zero (sodium $r = 0{\cdot}999$, potassium $r = 0{\cdot}999$, creatinine $r = 0{\cdot}999$, urea $r = 0{\cdot}999$), one channel was linear but not through zero (chloride $r = 0{\cdot}999$) and best fit for the bicarbonate channel was a quadratic through zero (bicarbonate $r = 0{\cdot}998$).

*Carry-over*

Carry-over effects as assessed by the method of Broughton *et al.* [4] could be minimized by employing the carry-over correction factor or by the use of curve regeneration. Carry-over correction factors were used for sodium, potassium and chloride channels and curve regeneration was employed for the remaining three channels (bicarbonate, urea, creatinine).

*Drift*

Table 4 shows the percentage drift present on samples over a 24 h period with and without the drift-correction program. The greatest drift present when the drift correction program was used was found to be with the bicarbonate samples (3·9%, i.e. 0·8 mmol/l at 20·8 mmol/l).

*Within-run imprecision*

Imprecision is very low on all channels. The coefficients of variation obtained were 47% of those specified by Technicon as acceptance criteria for their electrolyte autoanalysers (see table 5).

*Between-run imprecision*

The bicarbonate channel was the least precise channel with coefficients of variation of 5·92% at 19·92 mmol/l and 4·52% at 24·98 mmol/l. The coefficient of variations of all the other channels was very low with an average coefficient of variation of 1·59% (see table 6).

*Inaccuracy*

The assayed values and the consensus values were compared by regression analysis. The results shown are those obtained after

*Table 2. Concentration of aqueous standards used for calibration.*

| Standard No. | Sodium mmol/l | Potassium mmol/l | Chloride mmol/l | Bicarbonate mmol/l | Urea mmol/l | Creatinine μmol/l |
|---|---|---|---|---|---|---|
| 1 | 100 | 2 | 82 | 10 | 5 | 100 |
| 2 | 120 | 4 | 94 | 20 | 10 | 200 |
| 3 | 130 | 6 | 106 | 25 | 15 | 400 |
| 4 | 140 | 8 | 118 | 30 | 20 | 700 |
| 5 | 150 | 10 | 130 | 40 | 30 | 1000 |

*Table 3. Concentration of serum secondary standards used for calibration.*

| Standard No. | Sodium mmol/l | Potassium mmol/l | Chloride mmol/l | Bicarbonate mmol/l | Urea mmol/l | Creatinine μmol/l |
|---|---|---|---|---|---|---|
| 1 | 122 | 3·2 | 82 | 16 | 4·5 | 142 |
| 2 | 132 | 4·1 | 89 | 19 | 16·3 | 328 |
| 3 | 140 | 5·0 | 99 | 26 | 21·2 | 566 |
| 4 | 146 | 6·9 | 117 | 35 | 31·0 | 655 |

calibrating the instrument with secondary standards (see table 7). All the channels except bicarbonate appear to be satisfactory (bicarbonate $r = 0{\cdot}96$).

### *Problems during the evaluation period*

Three channels: creatinine, bicarbonate and urea, all suffered from an accumulation of deposits within the manifold leading to partially blocked flow cells. In the case of the bicarbonate and urea channels no cleaning solvent used was found to be satisfactory and so the tubing and injection blocks had to be changed at intervals. The injection blocks were removed for cleaning. The orange deposits within the creatinine channel could be removed by pumping dilute acetic acid through the colour reagent line.

The autosampler failed on several occasions during the first few months. The fault was rectified by the manufacturer and no further trouble has been encountered.

Initially, the pump central spindle suffered from marked mechanical wear. The central spindles were subsequently changed for a more robust material and no further wear has been noticed.

The gear-boxes have proved to be the most troublesome items on the Chemispek. None of the original gear-boxes have lasted more than one year. They have all suffered from mechanical wear. The manufacturer is aware of this problem and a redesigned gear-box will be available in the near future.

The first sample introduction blocks were found to be poorly made—they fell apart with use. The problem was overcome on site by using a stronger glue. The manufacturer supplied more robust introduction blocks at a later date, which proved to be more reliable.

The only problems encountered with the microprocessor during evaluation were that abnormal peaks were occasionally flagged, but the results appeared acceptable. This occurred mainly on the sodium channel where the peak contained a small shoulder. More seriously, however, abnormal peaks were obtained on the chloride channel—these were not flagged and the results were erroneous.

These faults were identifiable by the operator by visually inspecting the peaks on the monitor and reanalysing suspect peaks where necessary. The frequency of these faults has fallen with time: unflagged peaks are experienced about once a month.

*Table 4. Percentage drift over 24 h.*

| | Without drift correction ≃ x̄ | % drift | With drift correction ≃ x̄ | % drift |
|---|---|---|---|---|
| Sodium | 137 mmol/l | 12·3 | 145 mmol/l | 1·3 |
| Potassium | 5·1 mmol/l | 15·0 | 5·1 mmol/l | 1·1 |
| Chloride | 100 mmol/l | 16·5 | 97 mmol/l | 1·9 |
| Bicarbonate | 25·2 mmol/l | 21·0 | 20·8 mmol/l | 3·9 |
| Urea | 30·0 mmol/l | 21·5 | 20·4 mmol/l | 1·4 |
| Creatinine | 400 μmol/l | 28·5 | 362 μmol/l | 0·5 |

*Table 5. Within-run imprecision of the Chemispek.*

| Test | Concentration | Units | Coefficient of variation | *N* | Technicon acceptance criteria for SMAII CV% |
|---|---|---|---|---|---|
| Sodium | 149·5 | mmol/l | 0·57 | 20 | 0·67 |
| | 140·9 | mmol/l | 0·56 | 20 | 0·71 |
| | 122·2 | mmol/l | 0·37 | 20 | 0·82 |
| Potassium | 7·95 | mmol/l | 0·60 | 20 | 0·90 |
| | 5·02 | mmol/l | 0·69 | 20 | 1·38 |
| | 3·03 | mmol/l | 0·49 | 20 | 2·3 |
| Chloride | 120·0 | mmol/l | 0·40 | 20 | 1·08 |
| | 106·4 | mmol/l | 0·53 | 20 | 1·22 |
| | 84·9 | mmol/l | 0·44 | 20 | 1·55 |
| Bicarbonate | 35·4 | mmol/l | 0·95 | 20 | 2·83 |
| | 31·5 | mmol/l | 0·87 | 20 | 3·16 |
| | 23·2 | mmol/l | 1·20 | 20 | 4·30 |
| Urea | 19·9 | mmol/l | 0·53 | 20 | 1·00 |
| | 10·2 | mmol/l | 0·76 | 20 | 1·96 |
| | 5·3 | mmol/l | 1·63 | 20 | 3·76 |
| Creatinine | 576 | μmol/l | 1·70 | 20 | 3·0 |
| | 346 | μmol/l | 1·90 | 20 | 3·0 |
| | 123 | μmol/l | 4·90 | 20 | |

*Table 6. Between-run imprecision at two levels on the Chemispek.*

| Channel | Units | *N* | x̄ | SD | CV% |
|---|---|---|---|---|---|
| Sodium | mmol/l | 107 | 141·2 | 1·39 | 0·98 |
| | mmol/l | 107 | 151·0 | 1·33 | 0·88 |
| Potassium | mmol/l | 107 | 4·91 | 0·06 | 1·22 |
| | mmol/l | 107 | 5·44 | 0·07 | 1·34 |
| Chloride | mmol/l | 107 | 93·8 | 1·39 | 1·48 |
| | mmol/l | 107 | 102·5 | 1·41 | 1·38 |
| Bicarbonate | mmol/l | 107 | 19·92 | 1·18 | 5·92 |
| | mmol/l | 107 | 24·98 | 1·13 | 4·52 |
| Urea | mmol/l | 107 | 12·17 | 0·22 | 1·81 |
| | mmol/l | 107 | 29·71 | 0·57 | 1·92 |
| Creatinine | μmol/l | 107 | 187·7 | 5·81 | 3·09 |
| | μmol/l | 107 | 371·6 | 6·60 | 1·78 |

*Table 7. Regression analysis of external quality assurance consensus value and the assayed value*

| Channel | Units | Range | *N* | Regression coefficients *r* | Slope | Intercept |
|---|---|---|---|---|---|---|
| Sodium | mmol/l | 113–154 | 35 | 0·99 | 0·98 | 2·53 |
| Potassium | mmol/l | 2·5–7·5 | 35 | 0·99 | 0·99 | 0·06 |
| Chloride | mmol/l | 81–118 | 35 | 0·98 | 1·04 | −5·07 |
| Bicarbonate | mmol/l | 11·1–20·1 | 21* | 0·96 | 1·06 | −0·6 |
| Urea | mmol/l | 3·5–30·4 | 35 | 0·99 | 1·00 | 0·15 |
| Creatinine | μmol/l | 77–774 | 35 | 0·99 | 0·99 | −3·23 |

* Only Wellcome quality assurance samples used.

### Conclusions

With the exception of gear-box faults, mechanical problems encountered during the evaluation period have been resolved. It is hoped that the redesigned gear-box will be more robust.

Despite these early problems the Chemispek has been shown to perform very well using well-known chemistry methods. Using only 140μl of sample for six chemistries it has proved to be fast (120 specimen/h), precise and accurate.

### References

1. Skeggs, L. T. and Hochestrasser, H., *Clinical Chemistry*, **10** (1964), 918–936.
2. Marsh, W. H., Fingerhut, B. and Miller, M., *Clinical Chemistry*, **11** (1965), 624–627.
3. Chasson, A. L., Grady, H. T. and Stanley, M. A., *American Journal of Clinical Pathology*, **35** (1961), 83–88.
4. Broughton, P. M. G., Gowenlock, A. H., McCormack, J. J. and Neill, D. W., *Annals of Clinical Biochemistry*, **11** (1974), 207–218.
5. McLelland, A. S., Fleck, A., and Burns, R. F., *Annals of Clinical Biochemistry*, **15** (1978), 12–17.

